# A bacterial negative transcription regulator binding on an inverted repeat in the promoter for epothilone biosynthesis

**DOI:** 10.1186/s12934-017-0706-9

**Published:** 2017-05-23

**Authors:** Xin-jing Yue, Xiao-wen Cui, Zheng Zhang, Ran Peng, Peng Zhang, Zhi-feng Li, Yue-zhong Li

**Affiliations:** State Key Laboratory of Microbial Technology, School of Life Science, Shandong University, Jinan, 250100 China

**Keywords:** Negative transcription regulator, Transcription inhibition, Epothilone synthesis, Inverted repeat sequence in promoter, Myxobacteria, Proteobacteria

## Abstract

**Background:**

Microbial secondary metabolism is regulated by a complex and mostly-unknown network of global and pathway-specific regulators. A dozen biosynthetic gene clusters for secondary metabolites have been reported in myxobacteria, but a few regulation factors have been identified.

**Results:**

We identified a transcription regulator Esi for the biosynthesis of epothilones. Inactivation of *esi* promoted the epothilone production, while overexpression of the gene suppressed the production. The regulation was determined to be resulted from the transcriptional changes of epothilone genes. Esi was able to bind, probably via the N-terminus of the protein, to an inverted repeat sequence in the promoter of the epothilone biosynthetic gene cluster. The Esi-homologous sequences retrieved from the RefSeq database are all of the Proteobacteria. However, the Esi regulation is not universal in myxobacteria, because the *esi* gene exists only in a few myxobacterial genomes.

**Conclusions:**

Esi binds to the epothilone promoter and down-regulates the transcriptional level of the whole gene cluster to affect the biosynthesis of epothilone. This is the first transcription regulator identified for epothilone biosynthesis.

**Electronic supplementary material:**

The online version of this article (doi:10.1186/s12934-017-0706-9) contains supplementary material, which is available to authorized users.

## Background

Many kinds of microorganisms, such as actinomycetes, bacilli and myxobacteria, are excellent producers of secondary metabolites with various biological activities, potentially intriguing in anti-infection, anti-cancer and other pharmaceutical applications. The production of secondary metabolites in microorganisms is normally limited, probably due to the complex and mostly-unknown network of global and pathway-specific regulators, as well as the large biosynthetic gene cluster containing many gene modules [1–4]. Myxobacteria are able to produce diverse secondary metabolites [5–8]. Although a dozen gene clusters for the biosynthesis of secondary metabolites have been reported in myxobacteria, a few regulation factors have been identified. StiR is the first regulator identified for secondary mechanism in myxobacteria, which was found involving in the positive regulation of stigmatellin production in *Cystobacter fuscus* Cb f17.1 [9]. ChiR was found to serve as a pleiotropic regulator, which not only positively regulated the chivosazol biosynthesis, but also affected the fruiting body development in *Sorangium cellulosum* So ce56 [10]. NtcA, a famous global transcriptional regulator responsive to the concentration of nitrogen, was reported to be able to regulate the biosynthesis of chivosazol and etnangien negatively in *S. cellulosum* So ce56 [11].

Epothilones are originally produced by some strains of *S. cellulosum*, and are a group of antitumor compounds mimicking paclitaxel in the polymerization of tubulin [6, 12]. Epothilones are potential anticancer agents by their greater water solubility and efficacy against paclitaxel-resistant tumors [13, 14]. Researchers tried many methods to improve the epothilones yield, including co-cultivation of different strains [15], mutation and high-throughput screening of high-producing strains [16], immobilization of strains [17], heterologous expressions [18–24] and manipulation of related genes [25, 26]. However, the yield is still limited. The biosynthetic gene cluster of epothilone is a large operon of approximately 56 kb in size, containing seven homodromous open reading frames (ORFs) [27, 28]. Because of the lack of efficient protocols for genetic manipulation in *S. cellulosum*, the regulation mechanisms of this big operon have not yet been investigated and thus remains mostly unknown.

A key regulatory step that modulates bacterial gene expressions is the promoter recognition by RNA polymerase and transcription initiation [29]. A transcription factor can increase the rate of transcription initiation (activators) or prevent RNA polymerase from initiating transcription (repressors). Many repressors prevent transcription by binding DNA at positions directly interfering the binding of RNA polymerases. Thus, in the promoters subject to repression, operator sequences for a repressor are often found to overlap or be immediately adjacent to the transcription start site [30]. For example, HrcA, a classic heat-shock regulator, binds to the controlling inverted repeat of chaperone expression (CIRCE) to regulate expressions of the downstream *groEL*-*ES* operon and dnaK gene [31–33]. Transcription regulators achieve specific binding in normally a dimer or further multimer form; and most operators contain direct or inverted repeats of a 4–5 base pair sequence [34].

*Sorangium cellulosum* So0157-2 is an epothilone producing strain [35]. We previously sequenced the genome of So0157-2 [36], studied in details the characteristics of epothilone operon promoter in *Escherichia coli* (*E. coli*) [37], and randomly integrated the epothilone biosynthetic gene cluster into *M. xanthus* genome by transposition [22]. In this study, we reported the identification of a negative transcription regulator for the biosynthesis of epothilones.

## Results

### Inactivation of *esi* promotes the epothilone production in *S. cellulosum* So0157-2

**Fig. 1:**
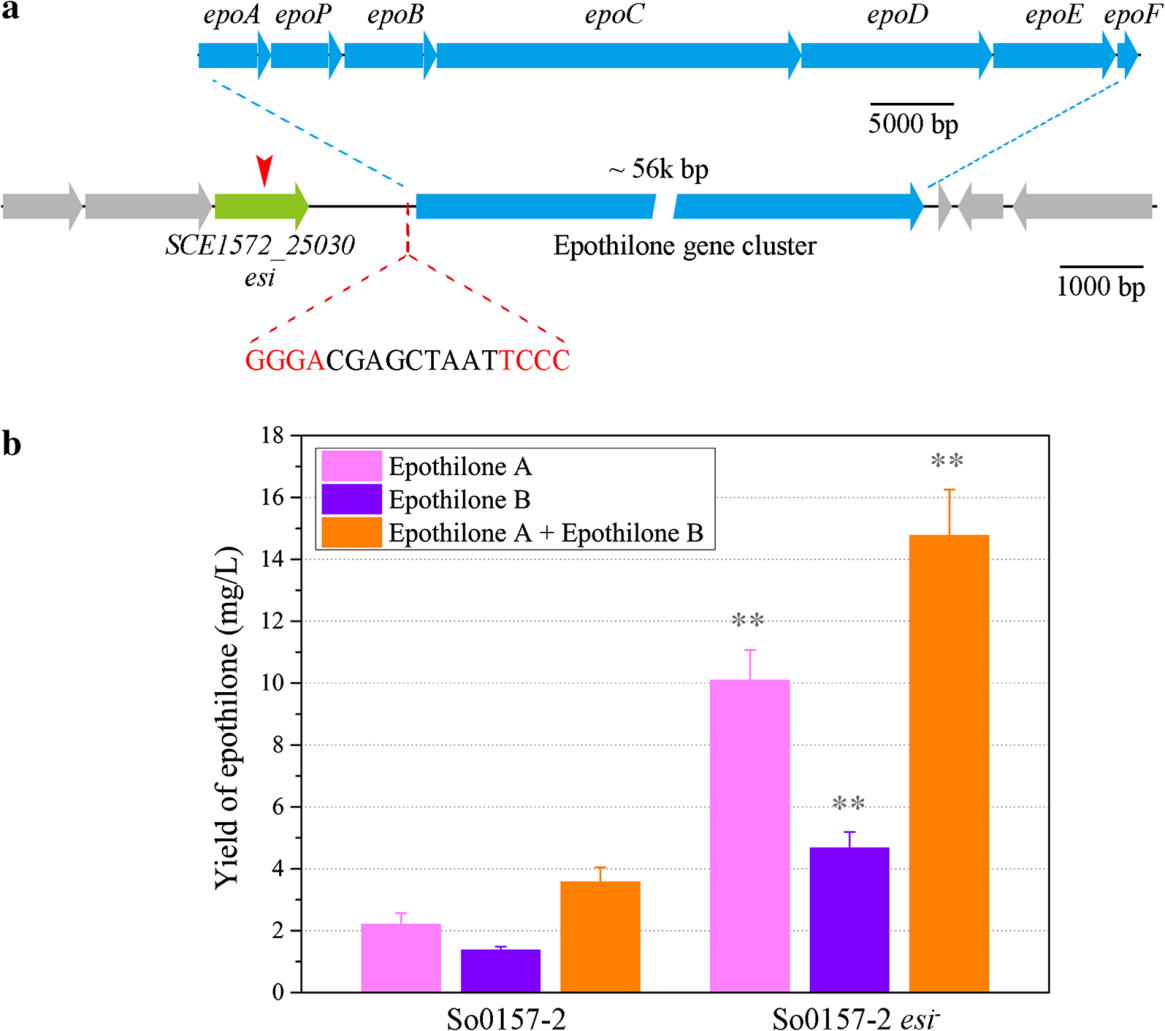
The biosynthetic gene cluster and productions of epothilones in *S. cellulosum* So0157-2 and So0157-2-*esi* ^−^. **a** A physical map of the epothilone gene cluster, showing the upstream *esi* gene *SCE1572_25030*, which was insertional inactivation (*red arrow*). The 17-bp sequence containing the four inverted repeat bases (in *red*) locates in the epothilone promoter. **b** Production of epothilones in So0157-2 and the *esi* ^−^ mutant in EMP medium. The *error bars* represent the standard deviation of three independent experiments. **P < 0.01

Using the conjugation and subsequent homologous recombination protocols as previously described [38], we inactivated genes flanking the epothilone gene cluster in *S. cellulosum* So0157-2 to determine whether they were associated with the synthesis, regulation or modification of epothilones. The *SCE1572_25030* gene locates approximately 500 bp upstream to the epothilone promoter (Fig. 1a). We amplified an internal fragment of the gene and cloned it into the pCC11 plasmid [38] to construct the inactivation vector, which was introduced into the genome of So0157-2. The gene was inactivated by insertional mutagenesis, which was confirmed by amplification using Tail-PCR and sequencing of the insertion region.

We fermented the mutant and the wild type strain in 50 mL of EMP medium supplemented with 2% of XAD-16 resin [16]. After 7 days of shaking cultivation, the resin was harvested, and the absorbed compounds were extracted for High Performance Liquid Chromatography (HPLC) analysis to determine the yields of epothilones. The results showed that the insertion mutation of *SCE1572_25030* resulted in a remarkable increase in the yields of epothilones A and B, from 2.2 and 1.4 mg/L in the wild type strain to 4.7 and 10.1 mg/L in the mutant (Fig. 1b; t test, *p* value <0.01). The total yields of epothilones A and B increased approximately 4.1 times, from 3.6 to 14.8 mg/L. The results suggested that the *SCE1572_25030* gene probably encoded an epothilone synthesis inhibitor (Esi). The mutant strain was termed as So0157-2-*esi* ^−^.

### The presence of *esi* gene suppresses the epothilone production in *M. xanthus*

**Fig. 2:**
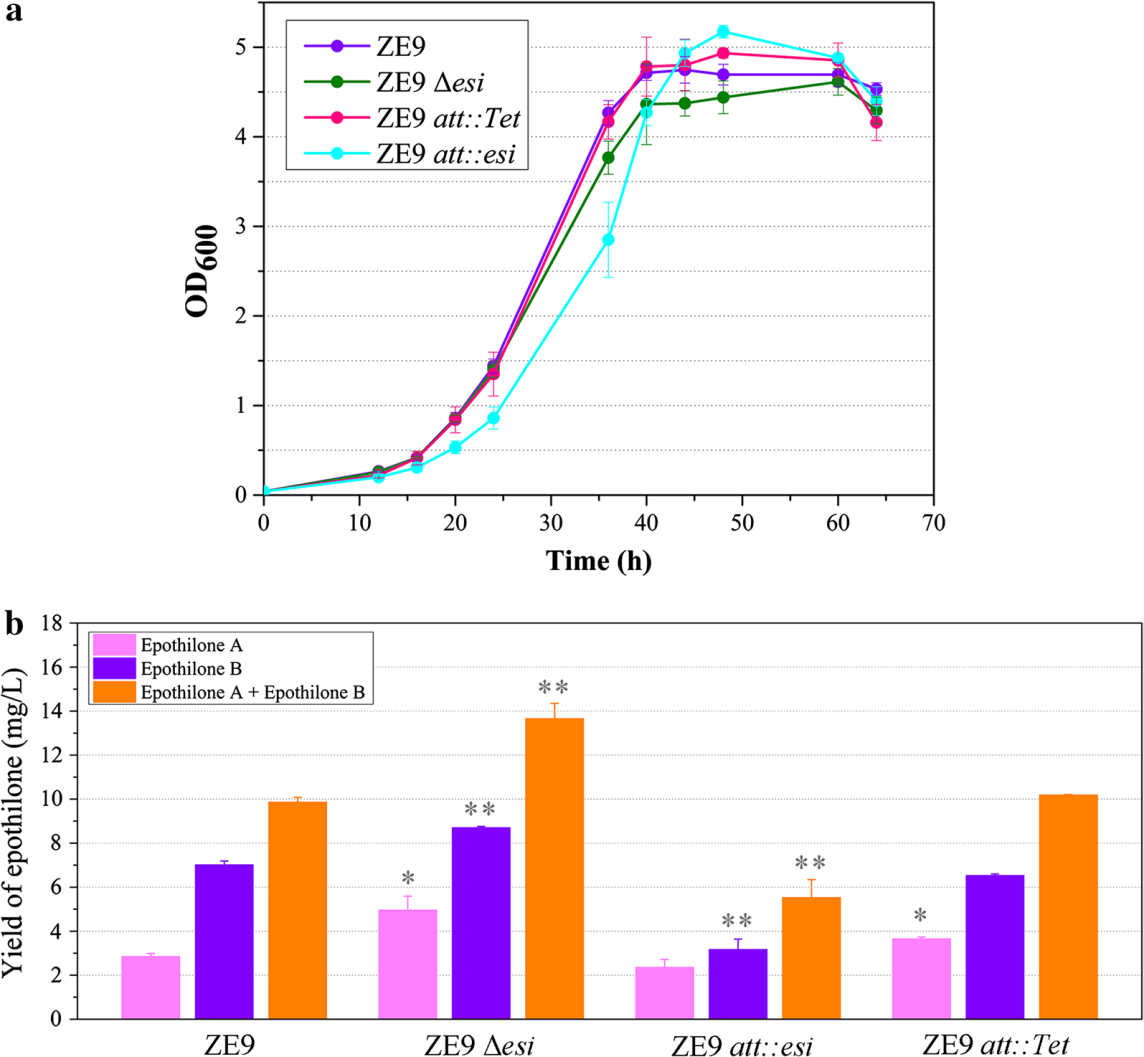
The growth curves (**a**) and production abilities of epothilones (**b**) in *M. xanthus* ZE9 and its mutants. The growth curves were assayed in CYE medium. The production of epothilones were detected in the CYE medium supplemented with methyl oleate (CMO medium). The *error bars* represent the standard deviation of three independent experiments. **P < 0.01 and *P < 0.05, respectively

Due to the genetic manipulation difficulty in *S. cellulosum* cells, we investigated influences of the *esi* gene on the production of epothilones in *M. xanthus*. We previously integrated the So0157-2 epothilone biosynthetic gene cluster randomly into *M. xanthus* genome by transposition; and the *esi* gene upstream to the epothilone gene cluster was also included in the transferred fragment [22]. We chose the ZE9 transformant derived from *M. xanthus* DZ2 for the assay. Bioinformatics analysis indicated that there was no homologous sequence of *esi* in DZ2, which was further confirmed by PCR amplification of an *esi* fragment against the DZ2 genome. Firstly, we deleted the *esi* gene from ZE9, producing the knockout mutant ZE9Δ*esi*. The ZE9 and ZE9Δ*esi* strains had similar growth curves in CYE medium (Fig. 2a). Methyl oleate is able to greatly increase the epothilone production ability in *Myxococcus* cells [39]. When fermented in the CYE medium supplemented with methyl oleate (CMO medium), the two strains showed different production abilities of epothilones. After 6 days of incubation, the yields of epothilones A and B increased 72.4 and 24.2%, and the total yields of epothilones were 9.9 mg/L in ZE9 and 13.7 mg/L in ZE9Δ*esi*, respectively (Fig. 2b). The deletion of *esi* significantly increased the production of epothilones in *Myxococcus* cells (*t* test, *p* value <0.01).

Furthermore, we constructed an *esi*-overexpressing mutant strain ZE9 *att*::*esi* by using plasmid pSWU30-p630-*esi* to introduce an additional *esi* gene into the *attB* site of ZE9 genome. Using the same process, the empty plasmid pSWU30-p630 was also introduced into ZE9, forming the ZE9 *att*::*Tet* strain as a control. These two strains were inoculated in CYE medium supplemented with 5 μg/mL tetracycline for 48 h, which were used as seeds to inoculate fresh CYE medium to assay their growth curves. The results showed that the ZE9 *att*::*esi* and ZE9 *att*::*Tet* strains also had a similar growth curve as the ZE9 strain (Fig. 2a). However, the introduction of an additional *esi* gene obviously suppressed the epothilone production (9.9 mg/L in ZE9 vs. 5.5 mg/L in ZE9 *att::esi*; *t* test, *p* value <0.01), whereas the ZE9 *att::Tet* had a similar epothilone yield as ZE9 (*t* test, *p* value >0.05) (Fig. 2b). Accordingly, Esi is a negative regulator for the production of epothilones, functioning not only in the producing *Sorangium* strains, but also in *Myxococcus*.

### Esi negatively regulates the transcription of epothilone biosynthetic genes

**Fig. 3:**
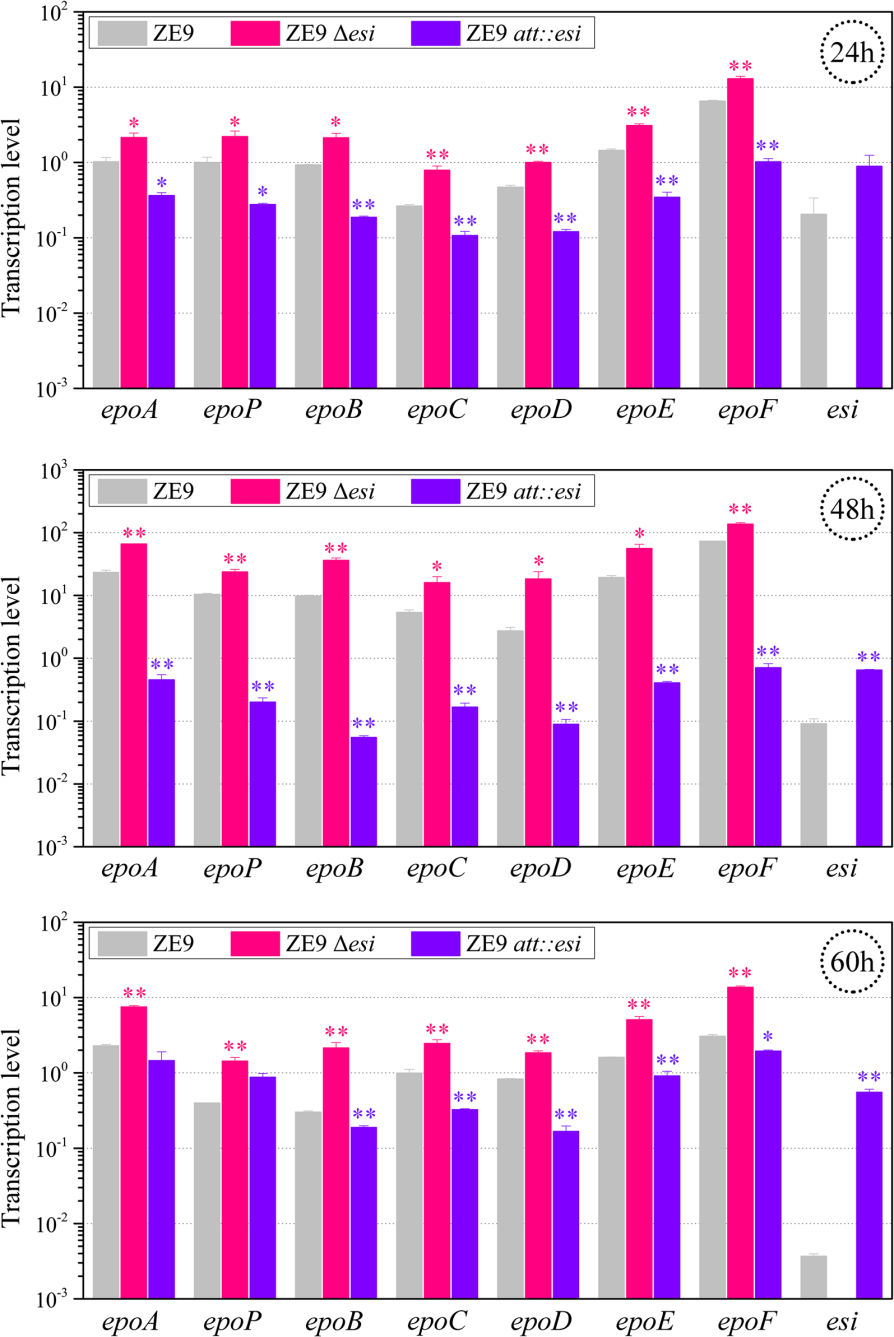
RT-qPCR analysis of expressions of the *esi* gene and the seven epothilone ORFs in the ZE9, ZE9 ∆*esi* and ZE9 *att*::*esi* strains after 24, 48 and 60 h of incubation in CMO medium. The expression level of *epoA* in the ZE9 strain at 24 h of incubation was set to be 1. The *error bars* represent the standard deviation of three independent experiments. **P < 0.01 and *P < 0.05, respectively

To investigate the regulation mechanism of Esi on the production of epothilones, we comparatively analyzed the transcriptional levels of epothilone biosynthetic genes in the *esi*-deletion and overexpression mutants with their parent strain *M. xanthus* ZE9 using quantitative real-time polymerase chain reaction (RT-qPCR). In ZE9 strain, compared with the expression of the *epoA* gene, the *esi* gene expressed at a low level (Fig. 3). In the early growth stage (24 h of incubation), expression of the *esi* gene was approximately 21% of the *epoA* gene; then weakly decreased with the increase of incubation. After the growth, the expression level of *esi* gene was greatly decreased, remaining less than 2% of the 24 h-expression at the 60 h of incubation (the stable stage of the growth curve, referred to Fig. 2b). Comparatively, in the ZE9 *att*::*esi* mutant, the additional *esi* gene was under the control of the 630-bp promoter of *pilA*, which held the total expression level of *esi* at high levels. The *esi* expressions at the 24, 48 and 60 h were 4.35-, 3.16- and 2.70-fold of that at the 24 h in the ZE9 strain (Fig. 3).

Similar to that in our previous report [22], transcriptional levels of the epothilone biosynthetic genes markedly varied in ZE9. With the increase of incubation time, transcriptions of the seven ORFs in the gene cluster each increased approximately ten times during the growth stage from 24 to 48 h, and then rapidly decreased in the following 12 h (Fig. 3). The transcriptional pattern was consistent with the production curves of epothilones in *M. xanthus* cells [22] or *S. cellulosum* cells [16], which was different from other microbial secondary metabolisms, usually expressed in stationary growth phase [2, 4].

After the deletion of the *esi* gene, the transcriptional level of each of the seven epothilone ORFs was markedly up-regulated at different incubation time points (*t* test, *p* value <0.05 or *p* value <0.01) (Fig. 3). The transcriptional increases ranged from 99% (*epoF*) to 200% (*epoC*) at 24 h, 88% (*epoF*) to 574% (*epoD*) at 48 h and 124% (*epoD*) to 611% (*epoB*) at 60 h of incubation. However, with the overexpression of *esi*, the epothilone genes were correspondingly down-regulated in the ZE9 *att::esi* strain (Fig. 3). The above RT-qPCR analysis demonstrated that Esi negatively regulated the transcription of epothilone genes.

### The structure model of Esi and its potential regulation mechanism

**Fig. 4:**
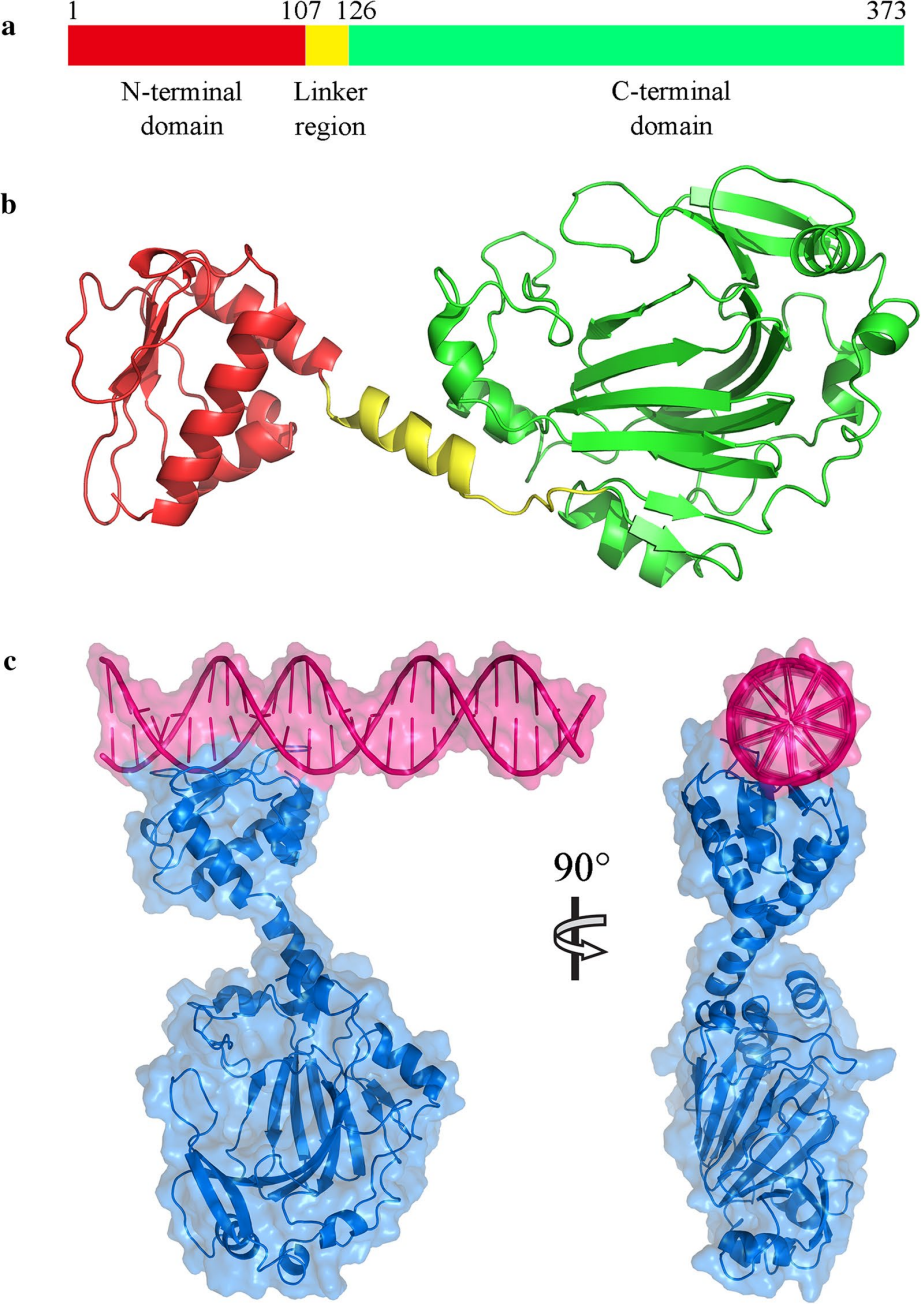
The structure modeling of Esi protein and the DNA-Esi binding complex. **a** Positions of N-terminal domain, linker region and C-terminal domain. **b** The structure model of Esi protein. The protein backbone is shown in Cartoon mode. There were three parallel β-pleated sheets and three alpha helixes in the N-terminal domain and an alpha helix in the linker region. The C-terminal region is a cupin-like domain. **c** The DNA–protein complex structure of Esi protein (*blue*) and epothilone promoter (*red*). The *top* of the N-terminal domain of Esi binds with the promoter DNA sequence

It is known that the repression of transcription initiation often occurs simply by steric hindrance [29]. From the above results, we suggested that the *esi* protein product probably functioned by directly binding on the epothilone promoter sequence to block the transcription. Esi (WP_020736929.1) is a protein with 373 amino acids in size. Domain prediction by the SMART program showed that the protein had no signal peptide or transmembrane domain. We constructed the three-dimensional (3D) structure of the Esi protein using the I-TASSER and QUARK programs. All the structural models were refined at the atomic level by using the fragment-guided molecular dynamics (FG-MD) simulations. Based on the constructed model, the Esi protein contained three regions, i.e. an N-terminal domain, a linker region and a C-terminal domain, with the isoelectric points of 6.90, 5.87 and 5.95, respectively (Fig. 4a). The 106-aa N-terminal domain had three parallel β-pleated sheets and three alpha helixes, the 248-aa C-terminal domain was a Cupin-like domain (pfam13621), and the linker region was an alpha helix (Fig. 4b). According to the quality assessment by Ramachandran plot (Additional file 1: Figure S1), the accuracy of protein structural models (80.6% of the residues in favored region) was acceptable [40].

The repressors, usually in dimer or further multimer form, bind to the operator sequence of promoter and thus block the transcription; and most operators contain direct or inverted repeats of a 4–5 base pair sequence for the binding [34]. We further constructed potential DNA–protein complex structure. We reconstructed a 3D structure of the epothilone promoter using w3DNA, and then calculated to determine the best complex structure of Esi-promoter by using nucleic acid-protein dock (NPDock). The results suggested that the N-terminal domain of Esi connected with the promoter DNA sequence (Fig. 4c). We checked the promoter sequence and found a special 17-bp DNA sequence (−137 to −153 bp relative to the transcriptional start site), which contains a 4-bp inverted repeat sequence (Fig. 1a). According to the DNA–protein complex structure, we suggested that the inverted repeat was the best Esi-binding region. The Esi protein might work as a dimer, and the two N-terminal domains bind specifically to the inverted GGGA of the double-strand promoter DNA sequence.

### Esi negatively regulates on epothilone promoter sequence

**Fig. 5:**
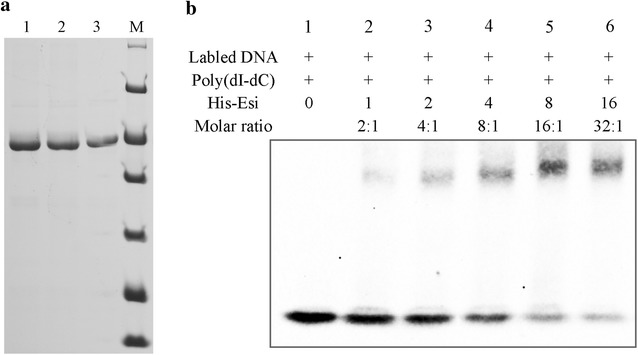
**a** The purified Esi protein with His-tag was detected by SDS-PAGE and visualized with Coomasie brilliant blue staining. *Lanes 1*, *2*, and *3* represented purified His-Esi protein; *Lane M* represented molecular mass standards (from *top* to *bottom*, 116, 66.2, 45, 35, 25, 18.4, 14.4 kDa). **b** EMSA showing the binding of His-Esi protein to the 17-bp operator DNA sequence of epothilone promoter. *Lane 1* represented free DNA with no His-Esi protein; *Lanes 2* to *6* represented DNA incubated with increased concentrations of His-Esi

In our previous report, we revealed that a 24-bp fragment in the epothilone promoter probably played a regulation role for the RNA polymerase functions [36]. Interestingly, this fragment includes the specific inverted repeat sequence. To determine the molecular mechanism of Esi regulation on the epothilone synthesis, we assayed the binding activity between Esi and the 17-bp operator DNA sequence of epothilone promoter. We constructed the *esi* gene into the expression vector pET-28a, forming pET-28a-*esi*, which was expressed in *E. coli* BL21 (DE3) cells. The Esi protein was tagged with a 6-His sequence. After induction with 0.1 mM IPTG, the Esi proteins were extracted from the cultivated cells, and purified based on the fused His-tag (Fig. 5a).

The double-strand 17-bp sequence was obtained by PCR annealing with the primers labeled with biotin. Electrophoretic Mobility Shift Assay (EMSA) was performed to detect the specific binding activity of Esi and the 17-bp sequence. Each of the reaction system contained biotin-labeled DNA and LightShift™ Poly (dI–dC). The results clearly showed that the His-Esi protein was able to bind to the biotin-labeled DNA. If the molar ratio of protein and DNA was increased, the more DNA was blocked (Fig. 5b), which indicated that the His-Esi protein bound specifically with operator DNA, rather than the nonspecific competition DNA (Poly dI–dC).

### Existence of *esi* gene in bacteria

Using the Esi amino acid sequence as a seed, we iteratively searched the homologous sequences in RefSeq database using the psi-BLAST program, and found 156 Esi-homologues with more than 90% of alignment coverage. The genes encoding for the 156 Esi homologous proteins distributed in 203 different bacterial genomes (Additional file 2: Table S1), all belonging to the Proteobacteria. These genomes contained single *esi* gene copy, except *Pseudomonas fluorescens* FW300-N2C3, which contained two genes encoding for the Esi homologous proteins. The *esi* gene distributed mainly in *Pseudomonas* (143 of the 203 genomes), *Acinetobacter* (41 genomes) and *Burkholderia* (6 genomes). Surprisingly, among more than 20 sequenced myxobacterial genomes, the *esi* gene was only found in the *Stigmatella aurantiaca* strain DW4/3-1 and the *S. cellulosum* strains So0157-2 and So0163. The results indicated that the Esi regulation mechanism was not universal in myxobacteria.

All of the Esi proteins had a length ranging from 356aa to 419aa, and their functions have not been reported, indicating that the *esi* gene codes a novel negative regulator. The evolutionary conservation of each amino acid residue in the Esi homologue proteins was calculated using ConSurf [41], which showed that the C-terminal domain had the highest conservation, followed by the N-terminal domain and the linker region (Additional file 3: Figure S2). Because of high similarities of their amino acid sequences, these homologous proteins were suggested to have similar 3D structures as the Esi protein described in this paper, probably playing functions as transcriptional regulation factors like the Esi protein.

## Conclusions

In this paper, we determined that the Esi protein played as a transcription regulator for epothilone biosynthesis. Inactivation of *esi* gene markedly promotes epothilone production. The Esi binds, probably via the N-terminus of the protein, to the inverted repeat of epothilone promoter and down-regulates the transcription of each gene in the gene cluster to suppress epothilones synthesis. Esi regulation is not universal in myxobacteria. This is the first transcription regulator identified for the biosynthesis of epothilones.

## Discussion

The Esi protein contained three regions, i.e. an N-terminal domain, a linker region and a C-terminal domain. The Esi protein connected with an operator of the promoter for the biosynthesis of epothilones, and the operator sequence contained a 4-bp inverted repeat in a 17-bp DNA fragment. We speculated that Esi worked in a similar way as HrcA [32, 33]: two molecules of Esi protein interacted to each other through the C-terminal domain, forming a mirrored dimer and the two N-terminal domains bind to the 4-bp inverted repeat of each DNA strand to repress transcription initiation. Although bacterial Esi proteins have not yet been studied, many of the homologous proteins were annotated to be cupin, a diverse superfamily of proteins named after its conserved barrel domain [42, 43] or transcription factor jumonji, a protein having a DNA binding domain and two conserved *jmj* domains [44]. The JmjC domains have been identified to play transcription factors in numerous eukaryotic proteins, such as PHD, C2H2, ARID/BRIGHT and zinc fingers, functioning by a histone demethylation mechanism that is conserved from yeast to human [44, 45]. However, since there is no histone in prokaryotes, the functioning mechanism of Esi was not like the eukaryotic JmjC proteins, and detailed molecular functioning pattern requires further investigations.

## Methods

### Bacterial strains, plasmids and culture conditions

Bacterial strains and plasmids used in this study are listed in Additional file 4: Table S2. *E. coli* strains XL1-Blue, DH5α λ pir and DH5α were used for cloning of pBJ113 plasmids, pSWU30 plasmids [46] and pET-28a plasmids, respectively. *E. coli* BL21 (DE3) was used to express the Esi protein. *E. coli* strains were grown at 37 °C in Luria-Broth (LB) medium (10 g/L peptone, 5 g/L yeast extract and 5 g/L NaCl, pH 7.2), and *M. xanthus* strains were grown at 30 °C in CYE medium [10 g/L casitone, 5 g/L yeast extract, 10 mM 3-(N-morpholino) propanesulfonic acid (MOPS) and 4 mM MgSO_4_, pH 7.6] or CMO medium [10 g/L casitone, 5 g/L yeast extract, 10 mM MOPS, 4 mM MgSO_4_ and 7 mL/L methyl oleate, pH 7.6]. The medium were supplemented with the following different antibiotics if required: ampicillin [Amp], 100 μg/mL; kanamycin [Km], 40 μg/mL; Apramycin, [Apra], 30 μg/mL, tetracycline [Tet], 5 μg/mL, and Gentamycin [Gm]. The primers used in constructing vectors and mutant strains were list in Additional file 5: Table S3.

### Inactivation of *esi* gene in So0157-2

Inactivation of *esi* was performed by insertion mutation. Using genomic DNA of So0157-2 as template, the homologous arm was amplified by PCR with primers *esi* F1 and *esi* R1. The PCR fragment was phosphorylated with T4 Polynucleotide Kinase (Fermentas, Canada) and then cloned into the SmaI restriction site of pCC11 [38], leading to the insertion plasmid pCC-*esi*. After verification by DNA sequence analysis, the pCC-*esi* was transformed into *E. coli* DH5α (*λ pir*) harbouring the pRK2003(Km^r^)plasmid, the resulting strain containing double plasmids was designed as ET-*esi*. The transformation of the plasmid pCC-*esi* into the genome of the So0157-2 was performed by biparental mating and conjugation, as described previously [38]. The *E.coli* ET-*esi* and So0157-2 were mixed with a ratio of 1:50, inoculated on the nitrocellulose membrane on VY/2 plate (7 μg/mL of Gm, 5 μg/mL of Cm), incubated for 40 h and then transferred to the selection VY/2 plate (15 μg/mL of Gm, 3 μg/mL of Cm). The clones appeared after two weeks of incubation at 30 °C were verified by Tail-PCR (data not shown) as described previously [22] and purified on CNST plate (15 μg/mL of Gm, 10 μg/mL of Cm). A Genome Walking kit (TaKaRa, Japan) was used to amplified the flanking sequence of insertion sites with three specific primers, Cm1, Cm2 and Cm3, designed in this work and a random primer AP1 provided by the kit. The PCR products with appropriate size from the third PCR round were withdrawn by the DNA Extraction kit (Promega, USA) and sequenced after cloning to the pMD19-T vector (Takara, Japan).

### Knockout and overexpression of *Esi* gene in ZE9

*Myxococcus xanthus* ZE9 was constructed with inserting the epothilone biosynthetic gene cluster and some flanking sequence, including the *esi* gene, into the genome of DZ2 by transposition in previous work [22]. The deletion of *esi* gene was performed by construction of knockout vector pBJ-*esi*. The arms for homologous recombination were amplified from the genome of So0157-2 by PCR using the primers *esi*-up F and *esi*-up R for upstream arm and *esi*-down F and esi-down R for downstream arm. The 1.6-Kb fragment of *esi*-ud was amplified by overlap PCR with *esi*-up mixed with *esi*-down as template and the primers *esi*-up F and *esi*-down R, digested with EcoRI and KpnI, and then cloned into pBJ113, constructing the knockout vectors pBJ-*esi*. The pBJ-*esi* was introduced into ZE9 by electroporation as described previously [22]. Resistant colonies that appeared after 6 days of incubation on CYE plates with 40 μg/mL of Km were checked by colony PCR with primers KG-test F and KG-trst R. The Km-resistant colonies were resuspended in 2.5 mL-tube with CYE liquid medium and diluted in gradient for 10^2^–10^5^ times, mixed with 2.5 mL of 0.5% soft agar and the mixture were spread on CYE selection agar plates containing 0.1% galactose to select the second recombination event. The galactose-resistant but Km-sensitive colonies were screened and checked by colony PCR with primers *Esi*-up F and *Esi*-down R. The knockout mutant, designated as ZE9∆*Esi*, was confirmed by sequence analysis of the PCR products.

To construct the overexpression mutant, the 630-bp promoter of the *pilA* gene (MXAN_RS28035) was amplified from the genomic DNA of DK1622 by PCR using the primers p630 F and p630 R. The PCR fragment was digested with HindIII and XbaI and then ligated into the plasmid pSWU30 [46], generating the vector of pSWU30-p630. The 1.2-Kb *esi* gene was amplified from the genomic DNA of So0157-2 by PCR with the primers *esi* F2 and *esi* R2 and ligated into XbaI and KpnI sites of pSWU30-p630 to produce the overexpression vector pSWU30-p630-*esi*. The pSWU30-p630-*esi* was electroporated into ZE9 and Tet-resistant colonies were screened by colony PCR using the primer p630 F and esi R2. The mutant strain ZE9 *att*::*Esi* was purified by being gradient diluted, mixed with soft agar (0.5%), and spread on CYE plates with 5 μg/mL of Tet. As a control, the empty plasmid pSWU30-p630 was also introduced into ZE9, producing ZE9 *att*::*Tet*.

### Transcriptional analysis of *esi* gene and epothilone genes

The mutant strains and wild type strains were cultured in 50 mL of CYE liquid medium for 20 h (OD600 was about 1.5), then transplanted into fresh CMO medium with a start OD600 of 0.04, and harvested at the early stage, middle stage and stable stage of exponential growth successively. Total RNA were extracted according to the protocol provided by the BIOZOL Toal RNA Extraction Regent (BioFast, China), and transcribed reversely into cDNA with PrimeScript™ Regent Kit with DNAase (Takara, Japan). We analyzed the relative transcriptional level of the *esi* gene and epothilone gene cluster by RT-qPCR on LightCycler^®^ 480 (Switzerland) with SYBR^®^ Premix Ex Taq™ GC Dye (Takara, Japan). The *gapA* gene (MXAN_RS13645), encoding glyceraldehyde-3-phosphate dehydrogenase, was used as the reference gene. The primers used in RT-qPCR were list in Additional file 6: Table S4.

### Extraction and detection of epothilones

So0157-2 and So0157-2-Esi^−^ were cultured in M26 medium (8 g/L potato starch, 2 g/L soy peptone, 2 g/L glucose, 2 g/L yeast powder, 1 g/L MgSO_4_, 1 g/L CaCl_2_, 1 mL/L EDTA-Fe^3+^, 1 mL/L microelement, pH 7.2) for 3 days and inoculated into 50 mL of EPM medium (4.7 g/L dextrin, 1.7 g/L soybean cake powder, 0.8 g/L glucose, 0.5 g/L saccharose, 2.2 g/L MgSO_4_·7H_2_O, 1 g/L CaCl_2_, 2 mL/L EDTA-Fe3^3+^, 1 mL/L corn steep liquor, 1 mL/L microelement, pH 7.5) supplemented with 2% of resin XAD 16 for the absorbtion of epothilone products. ZE9 and *esi* mutants were grown overnight in 50 mL of CYE medium supplemented with Apra (30 μg/mL). The cultures were inoculated at a ratio of 2:100 into 50 mL of CMO medium containing 2% of the XAD-16 resin. The resin were harvested with strainer after 9 days (for *S. cellulosum*) and 6 days (for *M. xanthus*) and extracted with 3 mL of methanol by shaking at room temperature overnight [16]. The supernatant was centrifuged for 10 min at 12,000 rpm and filtered with 0.22 µm filter to remove the impurities. 20 μL of the sample was injected into High Performance Liquid Chromatography (HPLC, SHIMADZU, Japan) and analyzed on a Shim-pack MRC-ODS RP C18 column (4.6 mm × 250 mm, 4.60 μm; Shimadzu, Japan) and monitored at 250 nm, with a mobile phase of 60% of methanol (HPLC grade) and 40% of H_2_O at a flow rate of 1.0 mL/min. The yield of epothilone was quantified from the peak area in the UV chromatogram, by reference against a calibration standard.

### Bioinformatics analysis of Esi

The signal peptides and transmembrane regions were predicted with SignalP [47] and TMHMM [48], respectively. Esi protein domain was annotated by SMART [49] and PFAM [50]. We constructed the N-terminal domain of Esi protein by ab initio protein folding and protein structure prediction with QUARK program [51], and then modeled the three-dimensional structures model of the whole Esi protein using the I-TASSER program based on a threading approach [52]. All the structural models were refined in the atomic-level by the fragment-guided molecular dynamics (FG-MD) simulations [53]. According to quality assessment by Ramachandran plot [40], the accuracy of the protein structural models were acceptable. The three-dimensional nucleic-acid structures of the epothilone promoter sequence was reconstructed with w3DNA server [54]. The NPDock (Nucleic acid-Protein Dock) was used to model the complex structures of Esi protein and epothilone promoter [55].

The position-specific iterative basic local alignment search tool (PSI-BLAST) [56] was used to search for homologous proteins of Esi in the US National Center for Biotechnology Information (NCBI) reference sequence (RefSeq) database [57]. The amino acid sequence of Esi protein was used as query sequence. A multiple sequence alignment of the Esi protein and its homologous proteins was established using the MAFFT program [58]. The evolutionary conservation of amino acid positions in the Esi protein and its homologous sequences was estimated by using ConSurf algorithm [41]. The JTT substitution matrix was used and the computation was based on the empirical Bayesian paradigm. The conservation scale was defined from the most variable amino acid positions (grade 1), which were considered to be evolved rapidly, to the most conservative positions (grade 9), which were considered to be evolved slowly. The sequence and modeled structure of the Esi protein were shown in nine-color conservation grades. The statistical analysis was conducted using IBM SPSS Statistics.

### Expression and purification of Esi protein in *E. coli*

Expression vector was constructed by amplifying the *esi* gene fragment, fused with a His-tag at N-terminus, with PCR using the primers of *esi* F3/*esi* R3 and ligating it into the XbaI and KpnI sites of the plasmid pET-28a, generating pET-28a-*esi*. *E. coli* BL21 (DE3) cells were transformed with plasmid pET-28a-*esi*. For the heterologous expression of Esi, 500 mL LB medium containing Km was inoculated with 5 mL of the overnight culture, prepared from a single colony, and grown at 37 °C for about 2 h. 0.1 mM IPTG was added at an optical density (OD600 nm) of 0.8. After 24 h of induced-expression at 16 °C, cells were harvested by centrifugation at 12,000 rpm for 5 min, resuspended with 1/10 volume of Lysis Buffer (25 mM Tris–HCl, 250 mM NaCl, pH 8.0) and disrupted by sonication. After centrifugation at 12,000 rpm for 30 min, the supernatant was incubated overnight with Ni–nitrilotriacetic acid HiszBind resin (Novagen), and then eluated with Lysis Buffer containing gradient imidazole ranging from 20 to 250 Mm to collect soluble purified Esi protein.

### Electrophoretic mobility shift assay

The biotin-labeled DNA fragment of operator DNA sequence was generated by PCR annealing (95 °C, 3 min; 90 °C, 1 min; 83 °C, 1 min; 76 °C, 1 min; 70 °C, 1 min; 65 °C, 1 min; 60 °C, 30 min) using the following oligonucleotides: 5′-GGGACGAGCTAATTCCC-3′ and 5′-GGGAATTAGCTCGTCCC-3′. The binding reactions were performed in 25 µL final volume containing 2.5 μL of reaction buffer (120 mM HEPES, 40 mM Tris, 600 mM KCl, 50 mM MgCl_2_, 1 mM EDTA, pH 8.0), 1 ul of glycerol buffer (20% glycerol in 20 Mm HEPES), 1 μL of biotin-labeled DNA fragment (12 pmol), 1 μL of Poly (dI–dC), N μL of protein and (17.5-N) μL of 50 mM NaH_2_PO_4_. The reaction samples were incubated at 30 °C for 30 min and then loaded into a 5% non-denaturing polyacrylamide gel to conduct electrophoresis with TGE buffer (14.3 g/L glycine, 3 g/L Tris, 1 mM EDTA, pH 8.0) for 2.5 h at 10 mA and 4 °C. The samples were electro-blotted onto an Amersham Hybond-N + membrane (GE, UK) by use of a mini transblot electrophoretic transfer cell (Bio-Rad). Hybridization and blotting was performed according to the protocol described by Chemiluminescent Nucleic Acid Detection Module (Thermo, USA). After completion of hybridization, the membrane was dried, autoradiographed, and the bands were detected by use of a Chemi Doc™ XRS + with Image Lab™ software (BIO-RAD).

## Additional files

**Additional file 1: Figure S1.** The ramachandran plot of the Esi structure model, which showed 80.6% residues in favored region, 13.5% residues in allowed region, 5.9% residues in outlier region.

**Additional file 2: Table S1.** Homologous amino acid sequences of Esi protein.

**Additional file 3: Figure S2.** Evolution conservative analysis of amino acid residues, calculated from the sequences of 156 Esi homologues. (A) Conservation scale is defined from the most variable residue sites (grade 1, color represented by turquoise; in rapid evolution) to conservative residue sites (grade 9, color represented by maroon; in slow evolution). (B) Conservation analysis of the Esi sequence sites in ConSurf grades.

**Additional file 4: Table S2.** Strains and plasmids used in this study.

**Additional file 5: Table S3.** Primers used in construction of vectors and mutant strains.

**Additional file 6: Table S4.** Primers used in RT-qPCR.
