# Supplementary figures and images for: A bacterial negative transcription regulator binding on an inverted repeat in the promoter for epothilone biosynthesis
Source: Microb Cell Fact. 2017 May 23;16:92. doi: 10.1186/s12934-017-0706-9 (PMC5442856; doi:10.1186/s12934-017-0706-9)

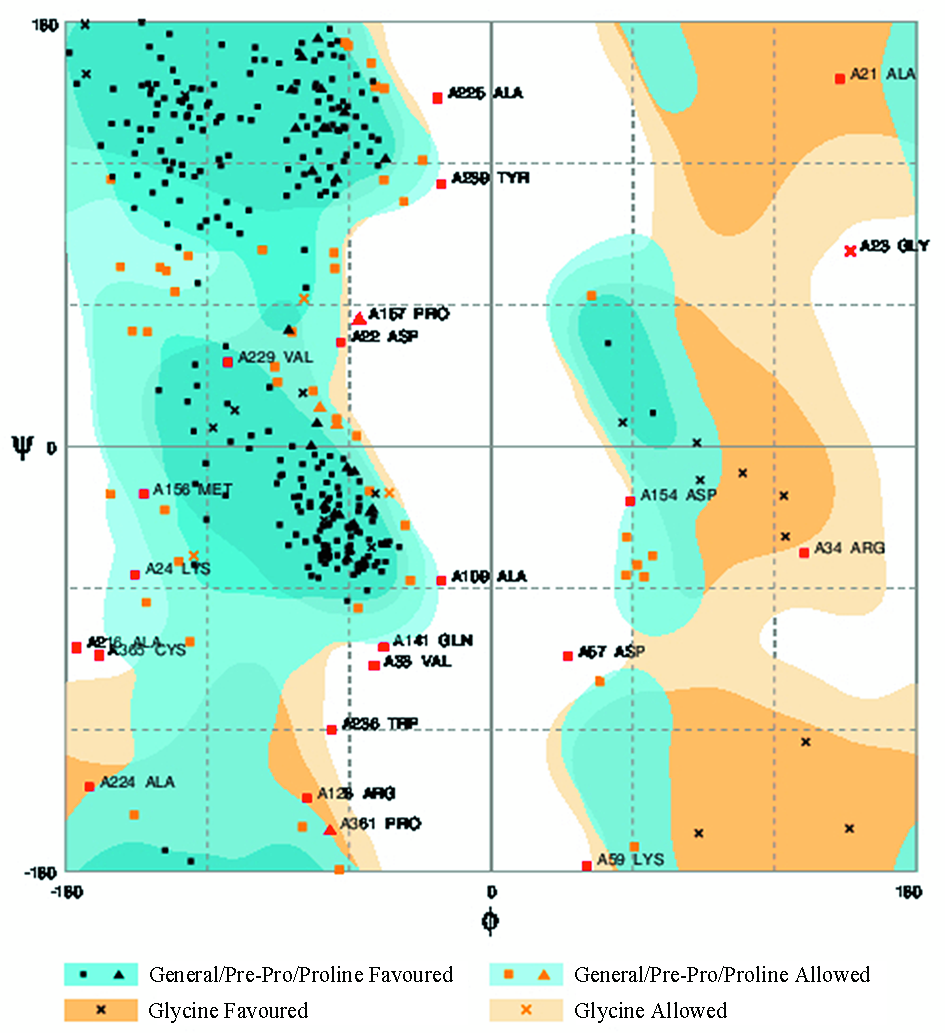

Supplement: Supplementary file 1 — Additional file 1: Figure S1. The ramachandran plot of the Esi structure model, which showed 80.6% residues in favored region, 13.5% residues in allowed region, 5.9% residues in outlier region. [file 12934_2017_706_MOESM1_ESM.tif]

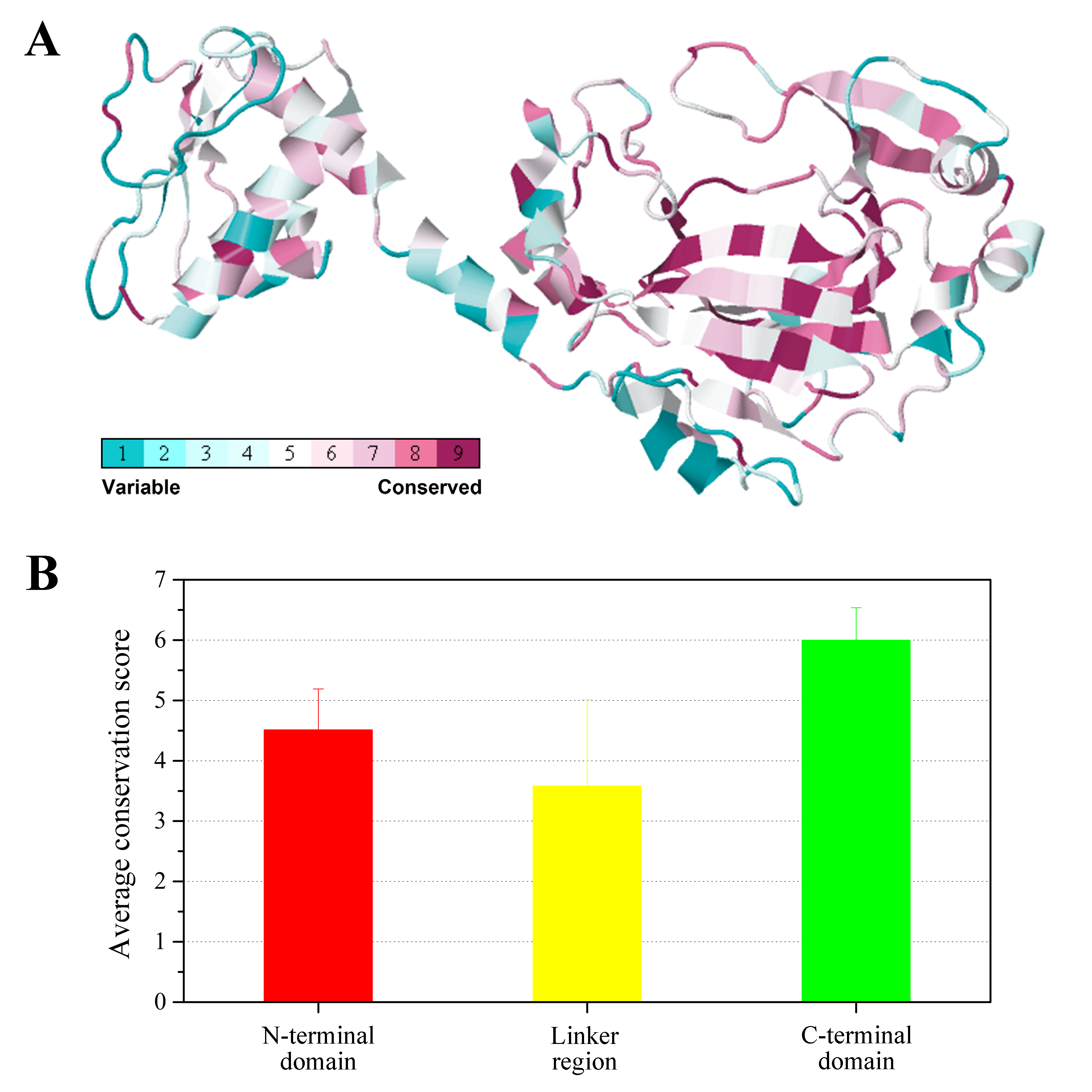

Supplement: Supplementary file 3 — Additional file 3: Figure S2. Evolution conservative analysis of amino acid residues, calculated from the sequences of 156 Esi homologues. (A) Conservation scale is defined from the most variable residue sites (grade 1, color represented by turquoise; in rapid evolution) to conservative residue sites (grade 9, color represented by maroon; in slow evolution). (B) Conservation analysis of the Esi sequence sites in ConSurf grades. [file 12934_2017_706_MOESM3_ESM.tif]
