# Supplementary material for: A bacterial negative transcription regulator binding on an inverted repeat in the promoter for epothilone biosynthesis
Source: Microb Cell Fact. 2017 May 23;16:92. doi: 10.1186/s12934-017-0706-9 (PMC5442856; doi:10.1186/s12934-017-0706-9)
Supplement: Supplementary file 2 — Additional file 2: Table S1. Homologous amino acid sequences of Esi protein. [file 12934_2017_706_MOESM2_ESM.docx]

**Table S1. Homologous amino acid sequences of Esi protein**

| **No.** | **Accession** | **Strain** | **Len**  **(aa)** | **Protein name** |
| --- | --- | --- | --- | --- |
| 1 | WP_020736929.1 | *Sorangium cellulosum* So0157-2 | 373 | hypothetical protein |
| 2 | WP_061622437.1 | *Sorangium cellulosum* So0163 | 373 | hypothetical protein |
| 3 | WP_032870731.1 | *Acinetobacter baumannii* 1000160 | 413 | cupin |
| 4 | WP_032870731.1 | *Acinetobacter baumannii* 1437282 | 413 | cupin |
| 5 | WP_032870731.1 | *Acinetobacter baumannii* 146457 | 413 | cupin |
| 6 | WP_016542424.1 | *Acinetobacter gyllenbergii* CIP 110306 | 413 | hypothetical protein |
| 7 | WP_016542424.1 | *Acinetobacter gyllenbergii* FMP01 | 413 | hypothetical protein |
| 8 | WP_016542424.1 | *Acinetobacter gyllenbergii* GTC 14627 | 413 | hypothetical protein |
| 9 | WP_016542424.1 | *Acinetobacter gyllenbergii* MTCC 11365 | 413 | hypothetical protein |
| 10 | WP_023270866.1 | *Acinetobacter gyllenbergii* NIPH 230 | 413 | hypothetical protein |
| 11 | WP_004957721.1 | *Acinetobacter junii* NIPH 182 | 414 | hypothetical protein |
| 12 | WP_050042151.1 | *Acinetobacter parvus* CM11 | 414 | cupin |
| 13 | WP_004674569.1 | *Acinetobacter parvus* NIPH 1103 | 404 | hypothetical protein |
| 14 | WP_070076398.1 | *Acinetobacter proteolyticus* ANC 3849 | 413 | cupin |
| 15 | WP_038344882.1 | *Acinetobacter* sp. A47 | 413 | cupin |
| 16 | WP_005218439.1 | *Acinetobacter* sp. ANC 3862 | 414 | hypothetical protein |
| 17 | WP_005315196.1 | *Acinetobacter* sp. ANC 3880 | 413 | hypothetical protein |
| 18 | WP_005155124.1 | *Acinetobacter* sp. ANC 3929 | 413 | hypothetical protein |
| 19 | WP_005192202.1 | *Acinetobacter* sp. ANC 4105 | 413 | hypothetical protein |
| 20 | WP_004762175.1 | *Acinetobacter* sp. CIP 102129 | 404 | hypothetical protein |
| 21 | WP_004800163.1 | *Acinetobacter* sp. CIP 102637 | 404 | hypothetical protein |
| 22 | WP_016164995.1 | *Acinetobacter* sp. CIP 110321 | 413 | hypothetical protein |
| 23 | WP_004802512.1 | *Acinetobacter* sp. CIP 56.2 | 413 | hypothetical protein |
| 24 | WP_005239888.1 | *Acinetobacter* sp. CIP 64.2 | 411 | hypothetical protein |
| 25 | WP_005200311.1 | *Acinetobacter* sp. CIP 70.18 | 413 | hypothetical protein |
| 26 | WP_033133262.1 | *Acinetobacter* sp. MN12 | 413 | cupin |
| 27 | WP_005239888.1 | *Acinetobacter* sp. NBRC 110496 | 411 | hypothetical protein |
| 28 | WP_005239888.1 | *Acinetobacter* sp. NCTC 7422 | 411 | hypothetical protein |
| 29 | WP_047431245.1 | *Acinetobacter* sp. neg1 | 413 | cupin |
| 30 | WP_005226645.1 | *Acinetobacter* sp. NIPH 1847 | 413 | hypothetical protein |
| 31 | WP_005278801.1 | *Acinetobacter* sp. NIPH 1859 | 411 | hypothetical protein |
| 32 | WP_005210326.1 | *Acinetobacter* sp. NIPH 1867 | 413 | hypothetical protein |
| 33 | WP_016653146.1 | *Acinetobacter* sp. NIPH 2036 | 411 | hypothetical protein |
| 34 | WP_005291605.1 | *Acinetobacter* sp. NIPH 2100 | 413 | hypothetical protein |
| 35 | WP_005255327.1 | *Acinetobacter* sp. NIPH 2168 | 413 | hypothetical protein |
| 36 | WP_004659090.1 | *Acinetobacter* sp. NIPH 236 | 414 | hypothetical protein |
| 37 | WP_005280659.1 | *Acinetobacter* sp. NIPH 3623 | 413 | hypothetical protein |
| 38 | WP_004774264.1 | *Acinetobacter* sp. NIPH 758 | 413 | hypothetical protein |
| 39 | WP_004656017.1 | *Acinetobacter* sp. NIPH 809 | 413 | hypothetical protein |
| 40 | WP_062848093.1 | *Acinetobacter* sp. NRRL B-65365 | 413 | cupin |
| 41 | WP_005200311.1 | *Acinetobacter* sp. TG19627 | 413 | hypothetical protein |
| 42 | WP_009514336.1 | *Acinetobacter* sp. WC-323 | 413 | cupin-like domain protein |
| 43 | WP_069579496.1 | *Acinetobacter* sp. YK3 | 413 | cupin |
| 44 | WP_012701115.1 | *Azotobacter* *vinelandii* CA | 375 | cupin |
| 45 | WP_012701115.1 | *Azotobacter* *vinelandii* CA6 | 375 | cupin |
| 46 | WP_012701115.1 | *Azotobacter* *vinelandii* DJ | 375 | cupin |
| 47 | WP_012701115.1 | *Azotobacter* *vinelandii* DSM 279 | 375 | cupin |
| 48 | WP_061289385.1 | *Azotobacter* *vinelandii* NBRC 13581 | 375 | cupin |
| 49 | WP_043283345.1 | *Burkholderia* sp. 2002721687 | 365 | transcription factor jumonji jmjC domain protein |
| 50 | WP_043283345.1 | *Burkholderia* sp. Bp5365 | 365 | transcription factor jumonji jmjC domain protein |
| 51 | WP_043283345.1 | *Burkholderia* *thailandensis* MSMB43 | 365 | transcription factor jumonji jmjC domain protein |
| 52 | WP_059716560.1 | *Burkholderia* *ubonensis* MSMB1172 | 354 | hypothetical protein |
| 53 | WP_059716560.1 | *Burkholderia* *ubonensis* MSMB1174WGS | 354 | hypothetical protein |
| 54 | WP_012487477.1 | *Cellvibrio* *japonicus* Ueda107 | 381 | N-acetyltransferase and transcription factor-like protein |
| 55 | WP_071898528.1 | *Cystobacter* *ferrugineus* Cbfe23 | 373 | cupin |
| 56 | WP_074295089.1 | *Paraburkholderia* *phenazinium* GAS95 | 419 | hypothetical protein |
| 57 | WP_028237330.1 | *Pseudomonas* *brassicacearum* 51MFCVI2.1 | 377 | cupin |
| 58 | WP_025214737.1 | *Pseudomonas* *brassicacearum* DF41 | 377 | cupin |
| 59 | WP_013693943.1 | *Pseudomonas* *brassicacearum* L13-6-12 | 377 | cupin |
| 60 | WP_013693943.1 | *Pseudomonas* *brassicacearum* LBUM300 | 377 | cupin |
| 61 | WP_047230457.1 | *Pseudomonas* *brassicacearum* LZ-4 | 377 | cupin |
| 62 | WP_013693943.1 | *Pseudomonas* *brassicacearum* NFM421 | 377 | cupin |
| 63 | WP_013693943.1 | *Pseudomonas* *brassicacearum* PA1G7 | 377 | cupin |
| 64 | WP_013693943.1 | *Pseudomonas* *brassicacearum* PP1_210F | 377 | cupin |
| 65 | WP_033699318.1 | *Pseudomonas* *capeferrum* WCS358 | 373 | cupin |
| 66 | WP_009044818.1 | *Pseudomonas* *chlororaphis* 30-84 | 378 | cupin |
| 67 | WP_038577462.1 | *Pseudomonas* *chlororaphis* *aurantiaca* JD37 | 377 | cupin |
| 68 | WP_023970436.1 | *Pseudomonas* *chlororaphis* *aurantiaca* PB-St2 | 377 | cupin |
| 69 | WP_038365342.1 | *Pseudomonas* *chlororaphis* EA105 | 377 | cupin |
| 70 | WP_062825173.1 | *Pseudomonas* *chlororaphis* isolate 189 | 378 | cupin |
| 71 | WP_075120788.1 | *Pseudomonas* *chlororaphis* PCL1601 | 377 | cupin |
| 72 | WP_047737818.1 | *Pseudomonas* *chlororaphis* UFB2 | 377 | cupin |
| 73 | WP_011533581.1 | *Pseudomonas* *entomophila* L48 | 389 | cupin |
| 74 | WP_029299843.1 | *Pseudomonas* *fluorescens* ATCC 17400 | 376 | cupin |
| 75 | WP_047291353.1 | *Pseudomonas* *fluorescens* AU5633 | 377 | cupin |
| 76 | WP_065257432.1 | *Pseudomonas* *fluorescens* BW11P2 | 377 | cupin |
| 77 | WP_013693943.1 | *Pseudomonas* *fluorescens* DSM 8569 | 377 | cupin |
| 78 | WP_058544468.1 | *Pseudomonas* *fluorescens* et76 | 377 | cupin |
| 79 | WP_014337392.1 | *Pseudomonas* *fluorescens* F113 | 378 | cupin |
| 80 | WP_060741389.1 | *Pseudomonas* *fluorescens* FW300-N2C3 | 377 | cupin |
| 81 | WP_060742326.1 | *Pseudomonas* *fluorescens* FW300-N2C3 | 374 | transcription factor jumonji jmjC domain protein |
| 82 | WP_034152816.1 | *Pseudomonas* *fluorescens* H16 | 377 | cupin |
| 83 | WP_042608397.1 | *Pseudomonas* *fluorescens* MEP34 | 377 | cupin |
| 84 | WP_039594561.1 | *Pseudomonas* *fluorescens* NT0133 | 377 | cupin |
| 85 | WP_016983509.1 | *Pseudomonas* *fluorescens* NZ011 | 377 | cupin |
| 86 | WP_011333307.1 | *Pseudomonas* *fluorescens* Pf0-1 | 376 | cupin |
| 87 | WP_030140864.1 | *Pseudomonas* *fluorescens* Pf29Arp | 377 | cupin |
| 88 | WP_003179636.1 | *Pseudomonas* *fluorescens* Q2-87 | 377 | cupin |
| 89 | WP_013693943.1 | *Pseudomonas* *fluorescens* Q8r1-96 | 377 | cupin |
| 90 | WP_024616036.1 | *Pseudomonas* *fluorescens* S12 | 377 | cupin |
| 91 | WP_039763052.1 | *Pseudomonas* *fluorescens* SF39a | 377 | cupin |
| 92 | WP_042732813.1 | *Pseudomonas* *fluorescens* UM270 | 377 | cupin |
| 93 | WP_013693943.1 | *Pseudomonas* *fluorescens* Wood1R | 377 | cupin |
| 94 | WP_039594561.1 | *Pseudomonas* *frederiksbergensis* SI8 | 377 | cupin |
| 95 | WP_027916318.1 | *Pseudomonas* *fulva* NBRC 16637 = DSM 17717 | 373 | cupin |
| 96 | WP_046063430.1 | *Pseudomonas* *kilonensis* 1855-344 | 377 | cupin |
| 97 | WP_053189540.1 | *Pseudomonas* *kilonensis* BS3780 | 377 | cupin |
| 98 | WP_041479067.1 | *Pseudomonas* *koreensis* D26 | 377 | cupin |
| 99 | WP_071171620.1 | *Pseudomonas* *koreensis* IMBL1 | 377 | cupin |
| 100 | WP_019579932.1 | *Pseudomonas* *mandelii* 36MFCvi1.1 | 377 | cupin |
| 101 | WP_028698358.1 | *Pseudomonas* *monteilii* IOFA19 | 373 | cupin |
| 102 | WP_061304257.1 | *Pseudomonas* *monteilii* MO2 | 373 | cupin |
| 103 | WP_024087422.1 | *Pseudomonas* *monteilii* SB3078 | 373 | cupin |
| 104 | WP_024087422.1 | *Pseudomonas* *monteilii* SB3101 | 373 | cupin |
| 105 | WP_042608397.1 | *Pseudomonas* *moraviensis* Devor | 377 | cupin |
| 106 | WP_024012350.1 | *Pseudomonas* *moraviensis* R28-S | 377 | cupin |
| 107 | WP_073474042.1 | *Pseudomonas* *moraviensis* UCD-KL30 | 377 | cupin |
| 108 | WP_058606007.1 | *Pseudomonas* *parafulva* NS212 | 373 | cupin |
| 109 | WP_058639692.1 | *Pseudomonas* *parafulva* NS96 | 373 | cupin |
| 110 | WP_027916318.1 | *Pseudomonas* *parafulva* YAB-1 | 373 | cupin |
| 111 | WP_023661380.1 | *Pseudomonas* *plecoglossicida* NyZ12 | 373 | cupin |
| 112 | WP_024087422.1 | *Pseudomonas* *plecoglossicida* TND35 | 373 | cupin |
| 113 | WP_062574727.1 | *Pseudomonas* *putida* 1A00316 | 381 | cupin |
| 114 | WP_046785273.1 | *Pseudomonas* *putida* ATH-43 | 376 | cupin |
| 115 | WP_019750826.1 | *Pseudomonas* *putida* B6-2 | 373 | cupin |
| 116 | WP_009394210.1 | *Pseudomonas* *putida* CSV86 | 378 | transcription factor jumonji, jmjC |
| 117 | WP_038410010.1 | *Pseudomonas* *putida* DLL-E4 | 373 | cupin |
| 118 | WP_012051711.1 | *Pseudomonas* *putida* F1 | 373 | cupin |
| 119 | WP_012273377.1 | *Pseudomonas* *putida* GB-1 | 373 | cupin |
| 120 | WP_054572529.1 | *Pseudomonas* *putida* HB13667 | 373 | cupin |
| 121 | WP_015271113.1 | *Pseudomonas* *putida* HB3267 | 373 | transcription factor jumonji domain-containing protein |
| 122 | WP_012273377.1 | *Pseudomonas* *putida* IAC-RBcr5 | 373 | cupin |
| 123 | WP_028698358.1 | *Pseudomonas* *putida* IOFA1 | 373 | cupin |
| 124 | WP_046615078.1 | *Pseudomonas* *putida* KG-4 | 373 | cupin |
| 125 | WP_021784832.1 | *Pseudomonas* *putida* LF54 | 373 | hypothetical protein |
| 126 | WP_003254249.1 | *Pseudomonas* *putida* LS46 | 373 | cupin |
| 127 | WP_016498671.1 | *Pseudomonas* *putida* NBRC 14164 | 373 | hypothetical protein |
| 128 | WP_012051711.1 | *Pseudomonas* *putida* ND6 | 373 | cupin |
| 129 | WP_047604120.1 | *Pseudomonas* *putida* PD1 | 373 | cupin |
| 130 | WP_012273377.1 | *Pseudomonas* *putida* PP112420 | 373 | cupin |
| 131 | WP_013973466.1 | *Pseudomonas* *putida* S16 | 373 | cupin |
| 132 | WP_023534020.1 | *Pseudomonas* *putida* S610 | 373 | jmjC domain, hydroxylase |
| 133 | WP_013973466.1 | *Pseudomonas* *putida* SF1 | 373 | cupin |
| 134 | WP_012051711.1 | *Pseudomonas* *putida* SJTE-1 | 373 | cupin |
| 135 | WP_039603606.1 | *Pseudomonas* *putida* SQ1 | 376 | cupin |
| 136 | WP_003254249.1 | *Pseudomonas* *putida* TRO1 | 373 | cupin |
| 137 | WP_012315381.1 | *Pseudomonas* *putida* W619 | 376 | cupin |
| 138 | WP_012051711.1 | *Pseudomonas* *putida* YKD221 | 373 | cupin |
| 139 | WP_070995033.1 | *Pseudomonas* sp. 06C 126 | 376 | cupin |
| 140 | WP_032862967.1 | *Pseudomonas* sp. 25 R 14 | 377 | cupin |
| 141 | WP_018925338.1 | *Pseudomonas* sp. 35MFCvi1.1 | 377 | cupin |
| 142 | WP_054051478.1 | *Pseudomonas* sp. 655 | 377 | cupin |
| 143 | WP_072392075.1 | *Pseudomonas* sp. 7SR1 | 377 | cupin |
| 144 | WP_061551074.1 | *Pseudomonas* sp. ABFPK | 373 | cupin |
| 145 | WP_039615129.1 | *Pseudomonas* sp. C5pp | 373 | cupin |
| 146 | WP_020300698.1 | *Pseudomonas* sp. CF161 | 376 | transcription factor jumonji, jmjC |
| 147 | WP_020289232.1 | *Pseudomonas* sp. CFII64 | 377 | transcription factor jumonji, jmjC |
| 148 | WP_018609406.1 | *Pseudomonas* sp. CFII68 | 377 | transcription factor jumonji, jmjC |
| 149 | WP_075045859.1 | *Pseudomonas* sp. DRA525 | 373 | cupin |
| 150 | WP_025339991.1 | *Pseudomonas* sp. FGI182 | 373 | cupin |
| 151 | WP_032862967.1 | *Pseudomonas* sp. FH4 | 377 | cupin |
| 152 | WP_007933123.1 | *Pseudomonas* sp. GM18 | 377 | cupin |
| 153 | WP_007956352.1 | *Pseudomonas* sp. GM25 | 377 | cupin |
| 154 | WP_007962115.1 | *Pseudomonas* sp. GM30 | 377 | cupin |
| 155 | WP_008013514.1 | *Pseudomonas* sp. GM50 | 377 | cupin |
| 156 | WP_008081745.1 | *Pseudomonas* sp. GM80 | 377 | cupin |
| 157 | WP_018925338.1 | *Pseudomonas* sp. GR 6-02 | 377 | cupin |
| 158 | WP_047581297.1 | *Pseudomonas* sp. H2 | 373 | cupin |
| 159 | WP_054617356.1 | *Pseudomonas* sp. In5 | 377 | cupin |
| 160 | WP_003254249.1 | *Pseudomonas* sp. JY-Q | 373 | cupin |
| 161 | WP_027592901.1 | *Pseudomonas* sp. LAIL14HWK12:I7 | 373 | cupin |
| 162 | WP_056836762.1 | *Pseudomonas* sp. Leaf127 | 374 | cupin |
| 163 | WP_056784287.1 | *Pseudomonas* sp. Leaf434 | 377 | cupin |
| 164 | WP_056801381.1 | *Pseudomonas* sp. Leaf58 | 373 | cupin |
| 165 | WP_064378798.1 | *Pseudomonas* sp. MS586 | 377 | cupin |
| 166 | WP_054889237.1 | *Pseudomonas* sp. NBRC 111118 | 373 | cupin |
| 167 | WP_012273377.1 | *Pseudomonas* sp. NBRC 111132 | 373 | cupin |
| 168 | WP_060546082.1 | *Pseudomonas* sp. NBRC 111136 | 373 | cupin |
| 169 | WP_003254249.1 | *Pseudomonas* sp. NBRC 111139 | 373 | cupin |
| 170 | WP_060494300.1 | *Pseudomonas* sp. NBRC 111140 | 373 | cupin |
| 171 | WP_072412217.1 | *Pseudomonas* sp. NFACC04-2 | 377 | cupin |
| 172 | WP_072392075.1 | *Pseudomonas* sp. NFACC09-4 | 377 | cupin |
| 173 | WP_072349293.1 | *Pseudomonas* sp. NFACC16-2 | 377 | cupin |
| 174 | WP_072392075.1 | *Pseudomonas* sp. NFACC43 | 377 | cupin |
| 175 | WP_072392075.1 | *Pseudomonas* sp. NFACC47-1 | 377 | cupin |
| 176 | WP_072459402.1 | *Pseudomonas* sp. NFACC49-2 | 377 | cupin |
| 177 | WP_063912740.1 | *Pseudomonas* sp. p21 | 373 | cupin |
| 178 | WP_041479067.1 | *Pseudomonas* sp. PTA1 | 377 | cupin |
| 179 | WP_030131468.1 | *Pseudomonas* sp. QTF5 | 377 | cupin |
| 180 | WP_016773891.1 | *Pseudomonas* sp. R62 | 377 | cupin |
| 181 | WP_047599112.1 | *Pseudomonas* sp. RIT288 | 377 | cupin |
| 182 | WP_064363891.1 | *Pseudomonas* sp. RIT-PI-o | 377 | cupin |
| 183 | WP_064389802.1 | *Pseudomonas* sp. RIT-PI-r | 377 | cupin |
| 184 | WP_057450174.1 | *Pseudomonas* sp. Root401 | 377 | cupin |
| 185 | WP_045487589.1 | *Pseudomonas* sp. StFLB209 | 375 | cupin |
| 186 | WP_027614203.1 | *Pseudomonas* sp. URIL14HWK12:I6 | 377 | cupin |
| 187 | WP_013693943.1 | *Pseudomonas* sp. URIL14HWK12:I7 | 377 | cupin |
| 188 | WP_027916318.1 | *Pseudomonas* sp. URMO17WK12:I11 | 373 | cupin |
| 189 | WP_023381045.1 | *Pseudomonas* sp. VLB120 | 389 | hypothetical protein |
| 190 | WP_041068241.1 | *Pseudomonas* sp. W15Feb9B | 377 | cupin |
| 191 | WP_047577751.1 | *Pseudomonas* *syringae* CEB003 | 377 | cupin |
| 192 | WP_032628716.1 | *Pseudomonas* *syringae* GAW0119 | 377 | cupin |
| 193 | WP_065836009.1 | *Pseudomonas* *syringae* GR12-2 | 377 | cupin |
| 194 | WP_052963089.1 | *Pseudomonas* *syringae* Riq4 | 377 | cupin |
| 195 | WP_027899647.1 | *Pseudomonas* *syringae* UB246 | 377 | cupin |
| 196 | WP_049818726.1 | *Pseudomonas* *taiwanensis* DSM 21245 | 389 | cupin |
| 197 | WP_053123581.1 | *Pseudomonas* *thivervalensis* LMG 21626 | 377 | cupin |
| 198 | WP_018925338.1 | *Pseudomonas* *umsongensis* 20MFCvi1.1 | 377 | cupin |
| 199 | WP_019579932.1 | *Pseudomonas* *umsongensis* UNC430CL58Col | 377 | cupin |
| 200 | WP_010196804.1 | *Psychrobacter* sp. PAMC 21119 | 402 | transcription factor jumonji domain-containing protein |
| 201 | WP_075102303.1 | *Psychrobacter* sp. Rd 27.2 | 402 | hypothetical protein |
| 202 | WP_028671068.1 | *Saccharospirillum* *impatiens* DSM 12546 | 370 | hypothetical protein |
| 203 | WP_002615601.1 | *Stigmatella* *aurantiaca* DW4/3-1 | 378 | cupin |
