# Supplementary material for: A bacterial negative transcription regulator binding on an inverted repeat in the promoter for epothilone biosynthesis
Source: Microb Cell Fact. 2017 May 23;16:92. doi: 10.1186/s12934-017-0706-9 (PMC5442856; doi:10.1186/s12934-017-0706-9)
Supplement: Supplementary file 4 — Additional file 4: Table S2. Strains and plasmids used in this study. [file 12934_2017_706_MOESM4_ESM.docx]

**Table S2. Strains and plasmids used in this study.**

| Strain and plasmid | | Relevant characteristics | | Source or reference |
| --- | --- | --- | --- | --- |
| Strain | | |  |  |
| *E.coli* | |  | |  |
| DH5α | | F−, *supE44, ΔlacU169 (ϕ80lacZΔM15), hsdR17, recA1, endA1, gyrA96, thi‑1, relA1* | | Life Technologies |
| DH5α (λ pir) | | DH5α containing  *λ pir* gene | | H.B. Kaplan |
| XL1-Blue MR | | *Δ(mcr A)183 Δ(mcrCB-hsdSMR-Mrr)173 endA1 supE44 thi-1 recA1 GYRa96 relA1 lac* | | Stratagene |
| BL21 (DE3) | |  | | Life Technologies |
| *S. cellulosum* | |  | |  |
| So0157‑2 | | Wild type, Epothilone producing strain | | This lab |
| So0157‑2 *esi*^-^ | | So0157‑2 carrying a mutation in *esi* gene by integration of pCC1-*esi* | | This study |
| *M. xanthus* | |  | |  |
| ZE9 | | A heterologous expressional host of epothilone, derived from *M. xanthus* DZ2 | | This lab |
| ZE9 △*esi* | | ZE9 with deletion of *esi* gene | | This study |
| ZE9 *att*::*Tet* | | ZE9 with integration of pSWU30-p630 | | This study |
| ZE9 *att*::*esi* | | ZE9 with integration of pSWU30-p630-*esi* | | This study |
| Plasmid | |  | |  |
| pCC11 | | pCVD442 inserted with *aphII* and *cat* | | (38) |
| pCC11-*esi* | | Ligating the homologous arm for insertion to the pCC1 | | This study |
| pBJ113 | | Gene replacement vector with KG cassette; Km^r^ | | Z.M. Yang, Virginia Tech |
| pBJ-*esi* | | Ligating the two homologous arms for deletion to the pBJ113 | | This study |
| pSWU30 | | Site specific integration vector with Mx8 attB integration site (Mx8); Tet^r^ | | (46) |
| pSWU30-p630 | | Ligating the promoter of *pilA* gene to the pSWU30 | | This study |
| pSWU30-p630-*esi* | | Ligating the *esi* gene to the pSWU30-p630 | | This study |
| pET-28a | | Expression vector | | Novagen |
| pET-28a-*esi* | | Ligating the *esi* gene to the pET-28a | | This study |
| pMD19-T | Universal vector | | Takara |  |
