# Supplementary material for: A bacterial negative transcription regulator binding on an inverted repeat in the promoter for epothilone biosynthesis
Source: Microb Cell Fact. 2017 May 23;16:92. doi: 10.1186/s12934-017-0706-9 (PMC5442856; doi:10.1186/s12934-017-0706-9)
Supplement: Supplementary file 5 — Additional file 5: Table S3. Primers used in construction of vectors and mutant strains. [file 12934_2017_706_MOESM5_ESM.docx]

**Table S3. Primers used in construction of vectors and mutant strains**

| Primer name | Sequence (5'-3') | Restriction Site | |
| --- | --- | --- | --- |
| *esi* F1 | CCGCAGACCGTCGTCCAGTTC | | None |
| *esi* R1 | CAGCATCTCACCGGCGTTCAGC | | None |
| Cm1 | CACATTCTTGCCCGCCTGAT | | None |
| Cm2 | GGCAATGAAAGACGGTGAGC | | None |
| Cm3 | TGCCGTCTGTGATGGCTTCC | | None |
| *esi*-up F | GGAATTCAGCCCGATCATCGCAGAGTAC | | EcoRI |
| *esi*-up R | GCGTGGCCTCGTGTCGAACC | | None |
| *esi*-down F | *GGTTCGACACGAGGCCACGC*CGGCGCGCGCAAGAACGTCG | | None |
| esi-down R | GGGGTACCTGCCAGCGAAAGTGACAAGG | | KpnI |
| KG-test F | GCTAGCCGAAATGACCGACCAAGC | | None |
| KG-test R | GGTGCCAGTGCGGGAGTTTCG | | None |
| p630 F | CCCAAGCTTCTGGCGAACTACTTCCTGTC | | HindIII |
| p630R | GCTCTAGAGGGGGTCCTCAGAGAAGGTT | | XbaI |
| *esi* F2 | GCTCTAGAATGCAATCTGAGCTACACAC | | XbaI |
| *esi* R2 | GGGGTACCCTACAGCAAGGTCGTCAAG | | KpnI |
| *esi* F3 | GGAATTCATGCAATCTGAGCTACACACGAACC | | EcoRI |
| *esi* R3 | CCCAAGCTTCTACAGCAAGGTCGTCAAGTCC | | HindIII |
