# Supplementary material for: A bacterial negative transcription regulator binding on an inverted repeat in the promoter for epothilone biosynthesis
Source: Microb Cell Fact. 2017 May 23;16:92. doi: 10.1186/s12934-017-0706-9 (PMC5442856; doi:10.1186/s12934-017-0706-9)
Supplement: Supplementary file 6 — Additional file 6: Table S4. Primers used in RT-qPCR. [file 12934_2017_706_MOESM6_ESM.docx]

**Table S4. Primers used in RT-qPCR**

| Primer name | Sequence (5’-3’) |
| --- | --- |
| *gapA* QF | GCCCTGGAAGAGCCTGAACG |
| *gapA* QR | TGGAGACGATGTGGTGCTTGG |
| *esi* QF | CCAGTTCACCGTTCGAGACA |
| *esi* QR | CGCCGTGGACAGGAAGAT |
| *epoA* QF | GCGTTCCACTCACCGCTCAT |
| *epoA* QR | GCCTTCCCGCTCAGATTGCT |
| *epoP* QF | GCTCAACATAACGCTCTTCAACC |
| *epoP* QR | CTGGACCTCGATACCGCTCA |
| *epoB* QF | ATGGAAGAACAAGATTCCTC |
| *epoB* QR | CTCGGAGAAGCGCTGCACGG |
| *epoC* QF | GAAGATGCGGTGAGGTTGGTGG |
| *epoC* QR | TCGGACGCTGCGATGGCTAC |
| *epoD* QF | GTGACAGACCGAGAAGGAC |
| *epoD* QR | CCACGATGGCGATCGGCTCG |
| *epoE* QF | GCACCGTTTGCGTTAGTAGGG |
| *epoE* QR | GCTTGGCTATTATGTCGGTCTCC |
| *epoF* QF | GGAGCAAGCGAATCAGAGTG |
| *epoF* QR | CGTGGTATCGGGTGAGGAC |
